# Physical punishment and effective verbal communication in children aged 9–36 months, according to sex: secondary analysis of a national survey

**DOI:** 10.1186/s12887-024-04606-4

**Published:** 2024-02-20

**Authors:** Vanessa Chire Illachura, Maria Inés Montesinos-Malpartida, Luciana Bellido-Boza, Zully M. Puyén, Dora Blitchtein-Winicki

**Affiliations:** Facultad de Ciencias de La Salud, Universidad Peruana de Ciencias Aplicadas, Av. Alameda San Marcos 11, Chorrillos, 15067 Lima, Perú

**Keywords:** Punishment, Communication, Infant, Child, Demographic health survey, Peru

## Abstract

**Background:**

A substantial number of children in the world are regularly subjected to physical punishment by their parents as a method of upbringing. Evidence suggests that it has negative effects on the development of brain function. However, evidence regarding its association with verbal communication is limited and heterogeneous. It is also unknown whether the effects are the same in both boys and girls; especially in the contexts of developing countries, where the highest rates of physical punishment are found.

**Objective:**

This investigation aimed at analyzing the association between physical punishment administered by both fathers and mothers and effective verbal communication among children aged 9–36 months according to sex.

**Methods:**

A secondary analytical cross-sectional study was conducted based on the 2018–2019 Peruvian Demographic and Family Health Survey. Physical punishment, based on the mother’s report of the use of hitting and/or slapping, was considered as a method to correct children by the father and/or mother. Effective verbal communication (EVC) was measured using the Battle scale which consists of age-appropriate questions included in the early childhood development module. A generalized linear model of the family and Log Poisson link option was used to identify the association between them, using the crude, general adjusted, and sex-stratified models.

**Results:**

Of all the children, 16.31% received physical punishment from their father and/or mother, wherein 16.65% were boys and 15.97% were girls. Moreover, 36.48% exhibited EVC, wherein 32.55% were boys and 40.50% were girls. Adjusting for socioeconomic level, witnessing violence, mother’s marital status, age, occupation, education level, language, number of children, and moderate-to-severe depressive symptoms, it was found that boys who received physical punishment from their father and/or mother have a 31% lower probability of EVC (adjusted prevalence ratio (aPR) 0.69, 95% confidence interval (CI) 0.58–0.83, *p* < 0.001), whereas no association was found in girls who received physical punishment from their father and/or mother and EVC (aPR 0.93, 95% CI 0.81–1.06, *p* = 0.278).

**Conclusions:**

An association was found between physical punishment administered by father and/or mother and reduced EVC among boys, whereas no such association was found among girls. It is possible that even though a significant impact has not been observed in girls during this early stage, they may experience consequences in later stages of life, further research is needed.

## Introduction

The early stages of a child’s life play a vital role in the formation of communication and language skills, which are crucial for their future cognitive abilities, expressive capabilities, and interpersonal interactions. These formative years also contribute remarkably to the development of a healthy sense of self-esteem and self-confidence. During this critical period, the child’s brain exhibits high adaptability and responsiveness to environmental stimuli; thus, leading to profound structural and functional changes [1, 2]. Notably, within the first 3 years of life, the brain undergoes remarkable transformations that are closely intertwined with the acquisition of verbal communication skills, encompassing the rapid assimilation of intricate linguistic aspects across diverse cultural backgrounds, specific phonetic variations, and sound mastery within the initial year. At approximately 6 months, the child demonstrates the ability to associate specific forms with their corresponding meanings, and by approximately 1 year, they begin uttering their initial words; thus, marking the commencement of rapid vocabulary expansion. By the age of 2, they are proficient in forming sentences and actively engage in conversations [1].

Effective verbal communication (EVC) encompasses a child’s capacity to express their knowledge, emotions, and thoughts. During the early stage of life—spanning from 9 to 36 months—a child must acquire the abilities to comprehend and produce sounds, grasp meanings, and acquaint themselves with conversations in their native language [3]. Sex-related disparities have been observed in EVC development, particularly concerning language acquisition during this stage. Dissimilarities arise in the development of the linguistic system, as approximately 70% of late talkers are boys, which also has implications on their overall proficiency in social communication. Notably, girls exhibit early development in certain communication aspects, including eye contact [4], utilization of gestures [5], imitation of gestures [6], joint attention [7], and social referencing [7]. Recent research has unveiled neurological differences related to language, originating in the neonatal period [8]. In response to speech stimuli, newborn females exhibit greater left-lateralized brain activity, whereas males demonstrate bilateral and simultaneous neural activations. These findings suggest an early maturation of language-related brain areas in girls, partially accounting for sex-based discrepancies in early language development and EVC capabilities [8]. However, some differences in EVC development are attributed to socioenvironmental learning that varies by gender and influences brain neuroplasticity [9, 10].

The development of language and EVC in the early years of life is a complex process, with adults playing a crucial role, and family context being of utmost importance [11]. Numerous factors contribute to the development of communication, including neuropsychological maturity, which can be influenced by birth asphyxia, infections, or inadequate nutrition. These health conditions are associated with structural and functional alterations in the brain. [11, 12]. Affection and cognitive development within the context of adult interaction are also pivotal factors. Disruptions in these domains such as lack of psychosocial stimulation or exposure to physical maltreatment (toxic stress), compromised brain structure, function, and development, consequently interferes with proper communication [11–13]. The consequences of altered factors influencing EVC can result in limitations in capacities and developmental potential from an early age, leading to disadvantages and obstacles throughout their lifespan. These repercussions extend to the child’s emotional, cognitive, physical, and mental well-being, across different developmental stages [12, 14]. It is estimated that approximately 250 million children under the age of 5 are at a risk of not reaching their full developmental potential due to adverse factors related to health, nutritional status, and psychosocial stimulation during early childhood. The majority of these children reside in developing countries, accounting for 43% globally [2].

Furthermore, it is during this stage of children’s lives that interaction with adults particularly parents occurs, with the aim of correcting and nurturing behaviors in education and child-rearing practices. Parents employ diverse methods influenced by their cultural and familial traditions, and a considerable portion resorts to physical discipline [15]. It is crucial to emphasize that this form of punishment is not intended to inflict harm or cause injuries [15, 16].

The majority of children worldwide live in countries where physical punishment is permitted. Roughly, 63% of children under the age of 5 (approximately 25 million) are subjected to regular physical punishment by their caregivers [16]. Several factors are associated with physical punishment in children under the age of 3. These factors include the child’s sex (more prevalent among males than females); younger maternal age; mothers with limited parenting experience; parents facing higher levels of stress, anxiety, and depression; parents who come from families that also utilized physical punishment as a disciplinary measure; residing in households with higher number of children; experiencing parental conflict; or living in single-parent families. An inverse relationship exists with socioeconomic status, wherein lower socioeconomic status is linked to a higher incidence of physical punishment. In addition, physical punishment rates vary among ethnic groups, with individuals of African descent exhibiting higher prevalence. Conversely, data on Hispanics is diverse across studies, showing higher, lower, or similar rates compared with other ethnicities, depending on migration circumstances and country-specific factors [15].

A substantial body of evidence links changes in brain structure and function to child maltreatment known as toxic stress. Similarities can be observed in the changes associated with physical punishment [13]. A recent study conducted during early childhood determined that physical punishment is associated with alterations in the neural processing of the threatening emotional stimuli. It is also associated with atypical structural and functional development in brain regions influenced by more severe forms of physical or sexual abuse (toxic stress), as shown by previous studies [13, 17, 18]. Moreover, severe physical punishment is linked to cognitive and behavioral deficits and changes in the brain’s processing of threatening emotional stimuli in children [17, 19]. Thus, physical punishment affects not only the body but also the brain and its functioning [13]. In fact, within the field of epigenetics, it has been established that experiences of violence during early childhood, such as maltreatment, elicit physiological changes. Prolonged exposure to such experiences leads to extended release of cortisol—the primary stress hormone—and DNA-level changes, thereby increasing the risk of psychiatric disorders, including post-traumatic stress disorder, anxiety, depression, and bipolar disorder in later stages of life [20].

The attention given to findings linking physical punishment to the consequences in cognitive development has been limited [15]. Moreover, few studies have determined its impact on cognitive abilities including communication with results displaying considerable heterogeneity. Out of the three studies, only one found an association between physical punishment and lower vocabulary scores [15], whereas the other two studies did not observe such a relationship [21, 22]. Despite the majority of children being born in developing countries (90%), the overwhelming majority of research on physical punishment and cognitive development encompassing verbal communication is conducted in developed countries. Consequently, studies examining this association in developing countries, where higher rates of physical punishment toward children have been identified, assume crucial importance [16, 23, 24]. The objective of this study is to evaluate the association between physical punishment—administered by both fathers and mothers—and EVC in children aged 9–36 months, stratified by sex, in Peru.

## Methods

This study employed a cross-sectional analytical design, conducting a secondary analysis of the 2018–2019 Demographic and Family Health Survey (ENDES) of Peru conducted by the National Institute of Statistics and Informatics (INEI). The ENDES sample was national representative, derived using a bi etapic, balanced, stratified, and independent probability design, covering urban and rural areas at the departmental level. This selection process involved cluster and rural census enumeration, with the primary sampling unit being the household.

### Population

The population for the current study consisted of children aged 9–36 months, whose mothers responded to modules inquiring about child discipline and early child development. Each mother’s youngest child was included. Exclusion criteria involved the absence of responses to questions regarding EVC or questions regarding physical punishment methods employed by the father and/or mother to correct the child.

### Study power

The power of the investigation was estimated using the OpenEpi software package, version 3.01, with a confidence interval (CI) of 95%. Referring to the study “Violence during Early Childhood and Child Development” [25], it was calculated that, by sex, 22.4% of children exposed to any type of violence exhibited adequate verbal communication effectiveness, whereas 77.3% of those not exposed also demonstrated adequate verbal communication effectiveness. With 3,722 nonexposed children to physical punishment and 692 who were, a power of more than 80% was calculated, even considering a design effect of two.

Regarding girls, the study revealed that 19.3% of those subjected to physical punishment exhibited adequate EVC, whereas 77.3% of those not exposed to violence also demonstrated the same. Considering 3,645 girls without any reported physical punishment and 631 who were, a power of more than 80% was calculated, even with a design effect of two.

### Variables

The dependent variable in this study is EVC, which is a part of the early childhood development module. The construction and validation of the communication domain involved the use of the battelle developmental inventory (BDI) as a psychological instrument. The BDI is suitable for assessing children from birth to 8 years of age and evaluates five developmental areas: cognitive, adaptative, motor, communication, and personal–social [26]. The reliability analysis of the EVC scale yielded a receptive reliability of 0.786 and an expressive reliability of 0.851 as measures of internal consistency [27]. The scale consists of four age groups: 9–12 months, 13–18 months, 19–23 months, and 24–36 months. Each age group is associated with four questions related to EVC. For the 9–12 months age group, the questions assessed word imitation upon hearing, understanding of meanings, comprehension of simple commands, and communication about current activities. In the 13–18 months group, the questions focused on word usage for making requests, object carrying orders, performing actions without demonstration, and communication about current activities. For the 19–23 months age range, the questions included naming body parts, word usage, obedience to complex commands, and participation in adult conversations. Lastly, the questions for the final age group (24–23 months) encompassed subject-action phrase usage, sentence construction, understanding of words indicating object positions, and engagement in adult conversations. If all questions within the respective age group received a “yes” response, it was classified as EVC. Otherwise, it was considered no EVC.

The independent variable in focus is physical punishment by the father and/or mother. The conflict tactics scale was employed as the assessment tool to evaluate this variable, demonstrating a Cronbach’s alpha of 0.62; thus, enabling the measurement of domestic violence [28]. Response categories within this scale encompass: slappings or hittings, verbal admonishments, privilege removal, food deprivation, physical punishment, confinement, ignoring, assigning the child additional tasks, evicting the child from the house, throwing water at the child, undressing the child, taking away the child’s belongings, economic withdrawal, burning, and wetting, among others [29]. For the purposes of this study, the responses considered were slappings and hittings as methods of correcting the child, specifically actions performed by the father and/or mother. Physical punishment was classified if the interviewee reported the use of slappings or hitting by either parent. If the child did not receive this form of punishment, it was categorized as no physical punishment.

Furthermore, the analysis took into account other variables, namely: socioeconomic level, determined by INEI through household characteristics and the availability of durable consumer goods using the Rutstein and Johnson methodology for wealth index calculation [30]. A score is generated for each household and subsequently computed as quintiles (poorest, poorer, middle, richer, and richest), age in months, sex and birth weight of the child, residential area, age in years, educational level, mother’s occupation and marital status, child’s primary caregiver, number of children, intimate partner violence, maternal language, and moderate-to-severe depressive symptoms in the mother measured using the Patient Health Questionnaire 9 (PHQ9) an instrument with solid evidence of validity and reliability to assess depressive symptoms over a span of 14 days [31, 32]. This instrument consists of nine questions that evaluate mood, sleep problems, fatigue, appetite changes, guilt, difficulty concentrating, and thoughts of death or suicide. Responses range from 0 to 3, with a maximum score of 27. The scores are classified into five categories, ranging from minimal to severe. In this study, a cutoff score of 10 was used to identify moderate to severe depressive symptoms, which are clinically significant and indicative of a higher risk for a major depressive episode [33].

### Study procedures

This study entails a secondary analysis of the 2018–2019 ENDES, freely available at https://proyectos.inei.gob.pe/microdatos/. A comprehensive database was created for the study, followed by quality control and statistical analysis. ENDES as the primary information source for our analysis collected data on our main variables through face-to-face interviews. The interviews were conducted using appropriate maternal language, respecting respondents’ cultural background, and emphasizing the confidentiality of their answers.

To gather the data of the early infant development module, trained ENDES INEI personnel, asked age-specific questions related to EVC to the child’s mother. In order to elicit spontaneous responses, the interviewers read the questions verbatim and waited to their instant answers. In addition, some questions included samples, in order to enhance comprehension in specific situations. Regarding questions about physical punishment, randomly selected women were interviewed according to the ENDES sampling system. At the start of the interview, the interviewer asked questions to verify privacy within the household, thus enabling the interview to proceed smoothly.

### Statistical analysis

Statistical analysis was conducted using the Stata 17 SE software package, with 95% confidence level.

In terms of descriptive analysis, categorical variables were presented using frequencies, weighted percentages, and 95% CI.

Regarding the bivariate analysis of categorical variables, Person’s chi-squares test with Rao–Scott corrects was applied.

Multivariable analysis involved the calculation of crude and adjusted models using the generalized linear framework with Log Poisson family and link option. The results were reported as prevalence ratios in a comprehensive model and two stratified models based on sex.

To incorporate confounding variables in the model, an epidemiological criteria was applied using a direct acyclic graph derived from a literature review [25, 34–38]. Collinearity analysis was conducted in the adjusted model using the variance inflation factor, where a value of 10 or above indicates multicollinearity. However, no variables exhibited multicollinearity. In addition, the correlation between independent variables was examined using the estat vce command, considering a correlation value greater than 0.5. A correlation was found between residential area and socioeconomic level, leading to the decision to include the latter variable (0.54).

The overall adjusted model included the following variables: sex, witnessing violence, socioeconomic level, marital status, mother’s age educational level, maternal language, number of children, moderate-to-severe depressive symptoms in the mother, and her occupation.

For the sex-stratified models, the following variables were considered as adjustment variables: socioeconomic level, witnessing violence, marital status, mother’s age, educational level, maternal language, number of children, moderate-to-severe depressive symptoms in the mother, and her occupation.

All analyses accounted for the sampling design using the svyset command, with the primary sampling unit being the stratum and the weight: svyset [pw = peso], psu (hv001) strata (hv022).

## Results

**Fig. 1:**
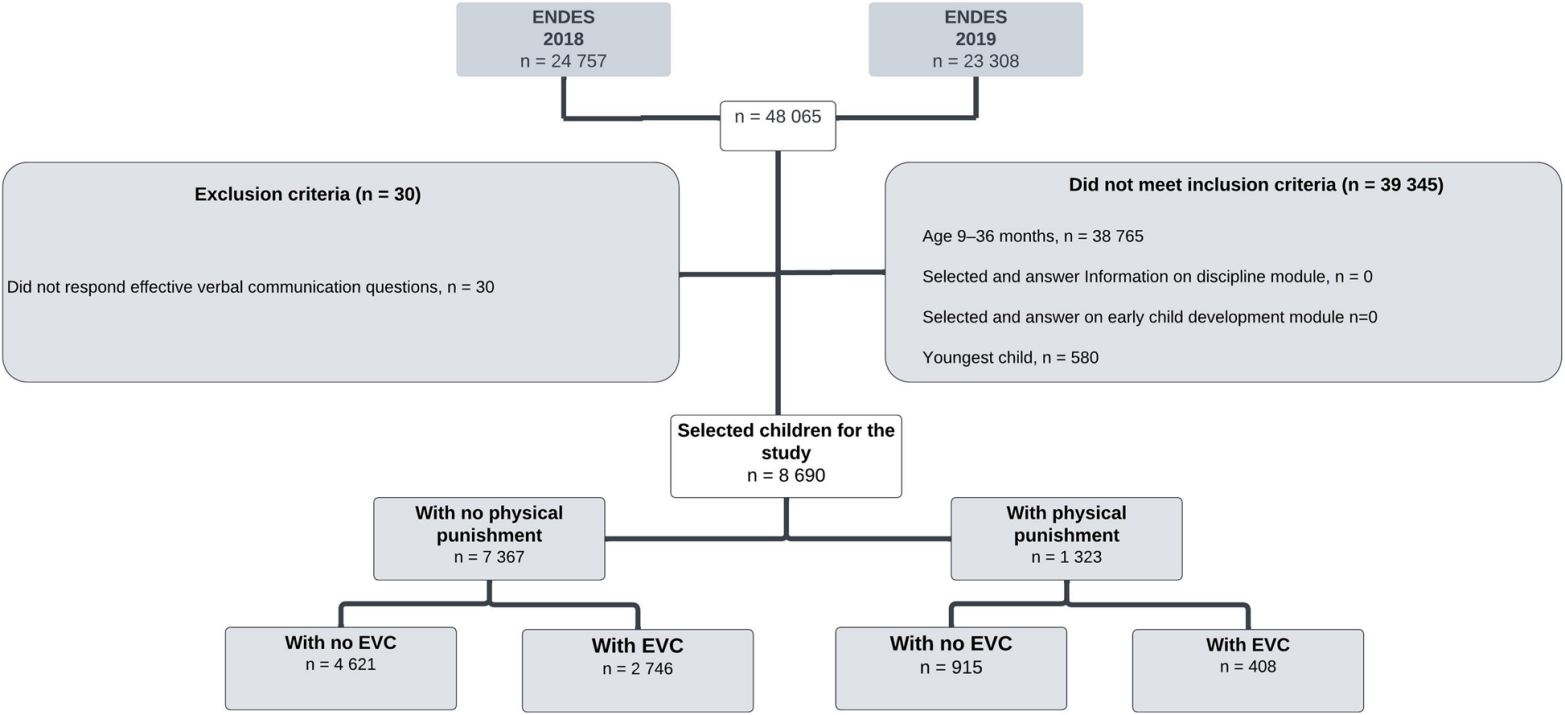
Boys and girls aged 9–36 months who met the study selection criteria

Within this study, a total of 8,690 children aged 9–36 months met the selection criteria (Fig. 1). Analysis of the data presented in Table 1 reveals that 45.05% of the children fell within the 24–36 months age range, and 50.56% were male. Approximately 71.97% of participants resided in urban areas, whereas 52.58% belonged to the two lowest socioeconomic levels: poorer and poorest. The primary caregiver for the child was the mother and/or her partner in 53.14% of cases. Regarding maternal education, the majority (65.35%) had completed secondary education or less. In terms of household dynamics, 21.43% of the children lived in households where the mother was a victim of partner violence. Moreover, 16.31% of the children experienced physical punishment from their parents, with 16.65% being boys and 15.97 being girls. Lastly, 36.48% of the children exhibited EVC skills, with 32.55% observed in boys and 40.50 in girls.

**Table 1:** Sociodemographic characteristics of the study on boys and girls aged 9–36 months in Peru, 2018–2019 (*n* = 8 690)

			95% CI	
	n	(%)^b^	LL	UL
**Characteristics**				
**Residential area**				
Urban	6038	(71.97)	70.83	73.09
Rural	2652	(28.03)	26.91	29.17
**Natural region**				
Coast	3422	(50.55)	49.11	51,99
Mountains	3070	(30.70)	29.18	32.26
Jungle	2198	(18.76)	17.50	20.08
**Socioeconomic level**				
Poorest	2538	(26.09)	24.90	27.32
Poorer	2486	(26.49)	25.22	27.80
Midlle	1678	(19.87)	18.75	21.03
Richer	1182	(15.17)	14.15	16.25
Richest	806	(12.39)	11.45	13.39
**Sex**				
Boys	4414	(50.56)	49.26	51.86
Girls	4276	(49.44)	48.14	50.74
**Age in months of the child**				
9–12 months	1311	(15.02)	14.13	15.96
13–18 months	1915	(22.51)	21.37	23.69
19–23 months	1531	(17.42)	16.44	18.45
24–36 months	3933	(45.05)	43.73	46.37
**Birth weight of the child** ^**c**^				
< 2500 g	507	(6.16)	5.52	6.87
≥ 2500 g	7940	(93.84)	93.13	94.48
**Principal caregiver**				
Mother	3521	(39.56)	38.21	40.93
Father	1193	(13.58)	12.91	14.76
Immediate family	3410	(40.94)	39.59	42.30
Other	566	(5.92)	5.33	6.57
**Violence between father and mother**				
There was no domestic violence	6772	(78.57)	77.47	79.63
Partner violence, but not a witness	1382	(15.17)	14.22	16.17
Witness partner violence	536	(6.26)	5.64	7.00
**Age of the mother**				
15–24 years	2098	(22.92)	21.84	24.04
25–34 years	4278	(49.61)	48.24	50.97
35–49 years	2314	(27.47)	26.23	28.75
**Level of education of the mother**				
Primary school or less	1764	(20.30)	19.28	21.52
Secondary school	4028	(45.05)	43.68	46.42
Higher nonuniversity	1583	(18.85)	17.79	19.96
University or higher	1315	(15.73)	14.68	16.83
**Marital status of the mother**				
Single	380	(4.27)	3.77	4.83
Married and cohabiting	7374	(85.37)	84.40	86.29
Widowed, divorced, and separated	936	(10.37)	9.58	11.22
**Maternal language**				
Spanish	6675	(81.01)	79.84	82.13
Indigenous and foreign languages	2015	(18.99)	17.87	20.16
**Currently working**				
No	3741	(43.84)	42.48	45.21
Yes	4949	(56.16)	54.79	57.52
**Number of children**				
One child	2598	(30.54)	29.28	31.82
Two children	2914	(33.57)	32.26	34.91
Three children	1688	(19.42)	18.34	20.54
Four or more children	1490	(16.47)	15.50	17.49
**Moderate-to-severe depressive symptoms in mother** ^**1**^				
No	8248	(94.72)	94.03	95.34
Yes	442	(5.28)	4.66	5.97
**Physical punishment by mother or father**				
No	7367	(83.69)	82.63	84.69
Yes	1323	(16.31)	15.31	17.37
**Effective verbal communication**				
No	5536	(63.52)	62.22	64.80
Yes	3154	(36.48)	35.20	37.78

*CI* 95% Confidence interval, *LL* Lower limit, *UL* Upper limit

^b^Weighted percentages

^c^243 missing values

^1^Criteria PHQ9 instrument: score ≥ 10

Table 2 displays the association between sociodemographic characteristics and EVC in children. It must be mentioned that there was a higher EVC in younger boys, specifically those aged 9–12 months compared with those aged 19–36 months (62.62% vs. 50.14% *p* < 0.001, respectively). In addition, boys residing in urban areas exhibited a higher EVC compared with boys in rural areas (34.32% vs. 28.04% *p* = 0.0004, respectively). Boys from the richest socioeconomic level demonstrated a higher EVC compared with those from the poorest level (40.13% vs. 26.81% *p* = 0.001, respectively). Moreover, boys whose mothers had achieved higher education levels, such as university or higher, had a higher EVC compared with those whose mothers had only completed primary education or less (40.50% vs. 28.12% *p* = 0.001, respectively). Lastly, boys from households with no violence had a higher EVC compared with those who witnessed violence at home (33.55% vs. 24.36% *p* = 0.025, respectively).

**Table 2:** Association between sociodemographic and study characteristics with effective verbal communication in boys aged 9–36 months. Peru, 2018–1019

**Characteristics**	**Effective verbal communication**						
	No			Yes			*p* ^e^
	*n* = 2983 (67.45%)			*n* = 1431 (32.55%)			
		95% CI			95% CI		
	n (%^a^)	LL	UL	n (%^a^)	LL	UL	
**Residential area**							
Urban	2038 (65.68)	63.54	67.76	1045 (34.32)	32.24	36.46	0.004
Rural	945 (71.96)	69.17	74.59	386 (28.04)	25.41	30.83	
**Natural region**							
Costal	1159 (65.57)	62.76	68.27	598 (34.43)	31.73	37.24	0.055
Mountain	1075 (69.12)	66.49	71.63	500 (30.88)	28.37	33.51	
Jungle	749 (69.81)	66.61	72.83	333 (30.19)	27.17	33.39	
**Socioeconomic level**							
Poorest	922 (73.19)	70.27	75.92	343 (26.81)	24.08	29.73	0.001
Poorer	865 (69.29)	66.10	72.25	403 (30.74)	27.75	33.90	
Middle	562 (63.97)	59.95	67.81	314 (36.03)	32.19	40.05	
Richer	391 (65.26)	60.60	69.64	205 (34.74)	30.36	39.40	
Richest	243 (59.87)	53.56	65.87	166 (40.13)	34.13	46.44	
**Age in moths of the boys**							
9–12 months	253 (37.38)	32.92	42.06	420 (62.62)	57.94	67.08	< 0.001
13–18 months	742 (76.21)	72.61	79.48	223 (23.79)	20.52	27.39	
19–23 months	654 (82.59)	79.03	85.65	132 (17.41)	14.35	20.97	
24–36 months	1334 (67.27)	64.70	69.73	656 (32.73)	30.27	35.30	
**Birth weight of the boys** ^**d**^							
< 2500 g	164 (67.31)	59.37	74.36	73 (32.69)	25.64	40.63	0.931
≥ 2500 g	2714 (66.96)	65.15	68.72	1334 (33.04)	31.28	34.85	
**Principal caregiver**							
Mother	1216(68.70)	66.71	71.29	564(31.30)	28.71	34.00	
Father	453 (73.10)	68.76	77.03	187 (26.90)	22.97	31.24	0.026
Immediate family	1116 (64.65)	61.72	67.48	580 (35.35)	32.52	38.28	
Other	198 (66.59)	59.39	73.09	100 (33.41)	26.91	40.61	
**Violence between father and mother**							
No domestic violence	2258 (66.45)	64.48	68.35	1154 (33.55)	31.65	35.52	0.025
Partner violence. but did not witness	503 (69.24)	64.74	73.4	208 (30.76)	26.60	35.26	
Witnessed intimate partner violence	222 (75.64)	69.06	81.20	69 (24.36)	18.80	30.94	
**Age of the mother**							
15–24 years	720 (69.43)	65.87	72.77	329 (30.57)	27.23	34.13	0.183
25–34 years	1454 (67.94)	65.53	70.26	695 (32.06)	29.74	34.47	
35–49 years	809 (65.00)	61.40	68.45	407 (35.00)	31.55	38.60	
**Level of education of the mother**							
Primary school or less	626 (71.88)	68.36	75.16	247 (28.12)	24.84	31.64	0.001
Secondary school	1400 (69.30)	66.73	71.75	631 (30.70)	28.25	33.27	
Higher nonuniversity	532 (65.09)	60.93	69.04	284 (34.91)	30.96	39.07	
University or higher	425 (59.50)	54.71	64.10	269 (40.50)	35.90	45.29	
**Marital status of the mother**							
Single	134 (67.56)	58.26	75.65	58 (32.44)	24.35	41.74	0.869
Married and cohabiting	2511 (67.27)	65.40	69.09	1221 (32.73)	30.91	34.60	
Widowed, divorced, and separated	338 (68.78)	63.40	73.71	152 (31.22)	26.29	36.60	
**Maternal language**							
Spanish	2280 (67.47)	65.52	69.36	1096 (32.53)	30.64	34.48	0.958
Indigenous and foreign languages	703 (67.36)	63.76	70.77	335 (32.64)	29.23	36.24	
**Currently working**							
No	1305 (68.77)	66.09	71.33	584 (31.23)	28.67	33.91	0.192
Yes	1678 (66.43)	64.12	68.66	847 (33.57)	31.34	35.88	
**Number of children**							
One child	873 (67.10)	63.81	70.23	425 (32.90)	29.77	36.19	0.857
Two children	1001 (67.81)	64.80	70.68	502 (32.19)	29.32	35.20	
Three children	575 (66.26)	62.16	70.14	262 (33.74)	29.86	37.84	
Four or more children	534 (68.65)	64.52	72.51	242 (31.35)	27.49	35.48	
**Moderate-to-severe depressive symptoms in mother** ^**1**^							
No	2813 (67.13)	65.34	68.87	1370 (32.87)	31.13	34.66	0.149
Yes	170 (72.78)	65.19	79.24	61 (27.22)	20.76	34.81	

*CI* 95% confidence interval, *LL* Lower limit, *UL* Upper limit

^a^All percentages are weighted

^d^129 missing values

^e^Pearson’s chi2 with Rao–Scott correction

^1^Criteria PHQ9 instrument: score ≥ 10

Table 3 presents the association between sociodemographic characteristics and EVC in girls. The results indicate a higher EVC in younger girls, specifically those aged 9–12 months compared with those aged 19–23 months (67.37% vs. 22.65% *p* < 0.001, respectively). In addition, girls residing in coastal areas showed a higher EVC compared with those in mountainous and jungle regions (43.92% vs. 37.84% y 35.78% *p* = 0.005, respectively). Girls from the richest socioeconomic level exhibited a higher EVC compared with those from the poorest level (46.47% vs. 32.44% *p* < 0.001, respectively). Moreover, girls whose mothers had achieved higher education levels, such as university or higher, had a higher EVC compared with those whose mothers had only completed primary education or less (48.85% vs. 31.71% *p* < 0.001, respectively). In addition, girls without siblings demonstrated a higher EVC compared with those with three, four, or more siblings (45.49% vs. 34.22% y 36.57 *p* = 0.001, respectively). Lastly, girls from households without violence had a higher EVC compared with those who witnessed violence at home but were not directly affected by it (41.88% vs. 34.19% *p* = 0.016, respectively).

**Table 3:** Association between sociodemographic characteristics of the study and effective verbal communication in girls aged 9–36 months. Peru, 2018–2019

**Characteristics**	**Effective Verbal Communication (EVC)**						
	No			Yes			p^e^
	*n* = 2553 (59.50%)			*n* = 1723 (40.50%)			
		95% CI			95% CI		
	n (%^a^)	LI	LS	n (%^a^)	LI	LS	
**Residence area**							
Urban	1789(57.35)	54.93	59.73	1246 (42.65)	40.27	45.07	0.001
Rural	844 (65.06)	61.92	68.08	477 (34.94)	31.92	38.08	
**Natural region**							
Costal	941 (56.08)	52.93	59.19	724 (43.92)	40.81	47.07	0.0005
Mountains	910 (62.16)	59.19	65.04	585 (37.84)	34.96	40.81	
Jungle	702 (64.22)	60.75	67.56	414 (35.78)	32.44	39.25	
**Socioeconomic level**							
Poorest	840 (67.56)	64.38	70.58	433 (32.44)	29.42	35.62	< 0.0001
Poorer	735 (58.88)	55.38	62.29	483 (41.12)	37.71	44.62	
Middle	446 (55.66)	50.97	60.25	356 (44.34)	39.75	49.03	
Richer	322 (56.50)	51.52	61.36	264 (43.50)	38.64	48.48	
Richest	210 (53.53)	47.31	59.63	187 (46.47)	40.37	52.69	
**Age in moths of the girls**							
9–12 months	202 (32.63)	28.40	37.16	436 (67.37)	62.84	71.60	< 0.0001
13–18 months	662 (69.78)	66.01	73.29	288 (30.22)	26.71	33.99	
19–23 months	588 (77.35)	73.16	81.05	157 (22.65)	18.95	26.84	
24–36 months	1101 (56.42)	53.52	59.27	842 (43.58)	40.73	46.48	
**Birth weight of the girls** ^**d**^							
< 2500 g	174 (65.15)	57.49	72.09	96 (34.85)	27.91	42.51	0.1019
≥ 2500 g	2297 (58.64)	56.60	60.65	1595 (41.36)	39.35	43.40	
**Principal caregiver**							
Mother	1051(59.51)	56.55	62.4	690(40.49)	37.60	43.45	
Father	326 (59.48)	54.07	64.67	227 (40.52)	35.33	45.93	0.4471
Immediate family	1025 (60.08)	57.01	63.07	689 (39.92)	36.93	42.99	
Others	151 (54.67)	46.68	62.42	117 (45.33)	37.58	53.32	
**Violence between mother and father**							
No domestic violence	1959 (58.12)	55.97	60.24	1401 (41.88)	39.76	44.03	0.0158
Partner violence. but did not witness	444 (65.81)	60.96	70.36	227 (34.19)	29.64	39.04	
Witnessed intimate partner violence	150 (61.42)	53.46	68.81	95 (38.58)	31.19	46.54	
**Age of the mother**							
15–24 years	642 (58.97)	55.08	62.74	407 (41.03)	37.26	44.92	0.9425
25–34 years	1263 (59.79)	56.99	62.52	866 (40.21)	37.48	43.01	
35–49 years	648 (59.42)	55.69	63.04	450 (40.58)	36.96	44.31	
**Level of education of the mother**							
Primary school or less	614 (68.29)	64.50	71.85	277 (31.71)	28.15	35.50	< 0.001
Secondary school	1195 (60.05)	57.21	62.81	802 (39.95)	37.19	42.79	
Higher Nonuniversity	425 (55.22)	50.65	59.71	342 (44.78)	40.29	49.35	
University or higher	319 (51.15)	45.48	56.79	302 (48.85)	43.21	54.52	
**Marital status of the mother**							
Single	115 (59.35)	50.41	67.71	73 (40.65)	32.29	49.59	0.301
Married and cohabiting	2185 (60.02)	57.94	62.07	1457 (39.98)	37.93	42.06	
Widowed, divorced, and Separated	253 (55.04)	48.81	61.12	193 (44.96)	38.88	51.19	
**Maternal language**							
Spanish	1934 (59.06)	56.87	61.22	1365 (40.94)	38.78	43.13	0.317
Indigenous and foreign languages	619 (61.39)	57.31	65.31	358 (38.61)	34.69	42.69	
**Currently working**							
No	1123 (60.54)	57.61	63.39	729 (39.46)	36.61	42.39	0.333
Yes	1430 (58.67)	56.13	61.17	994 (41.33)	38.83	43.87	
**Number of children**							
One child	720 (54.51)	51.06	57.92	580 (45.49)	42.08	48.94	0.001
Two children	835 (58.49)	55.07	61.83	576 (41.51)	38.17	44.93	
Three children	550 (65.68)	61.46	69.66	301 (34.22)	30.34	38.54	
Four or more children	448 (63.43)	59.04	67.61	266 (36.57)	32.39	40.96	
**Moderate-to-severe depressive symptoms in mother** ^**1**^							
No	2426 (59.50)	57.49	61.48	1639 (40.50)	38.52	42.51	0.977
Yes	127 (59.38)	50.87	67.36	84 (40.62)	32.64	49.13	

*CI *95% confidence interval, *LL *Lower limit, *UL *Upper limit

^a^ All percentages are weighted

^d^ 114 missing values

^e^ Pearson’s chi2 with Rao–Scott correction

^1^ Criteria PHQ9 instrument: score ≥ 10

As shown in Table 4, in the crude overall model for boys and girls, it was found that, compared with those who were not subjected to physical punishment by their father and/or mother, infants who experienced physical punishment had a 19% lower likelihood of having effective verbal communication (cPR 0.81 95% CI 0.73;0.91 *p* < 0.001). In the overall model adjusted for the sex of the child, witnessing violence, socioeconomic status, mother’s marital status, age, education level, maternal language, number of children, moderate-to-severe depressive symptoms in the mother, and her occupation, an 18% lower likelihood of children having EVC was found (aPR 0.82 95% CI 0.73;0.91 *p* < 0.001).

**Table 4:** Association between physical punishment by the father and/or mother and effective verbal communication in boys and girls aged 9–36 months; Peru 2018–2019 (*n* = 8 690)

**Effective verbal communication**	**General model for boys and girls** ^1^ **(** ***n*** ** = 8 690)**						**Boys **2** (** ***n*** ** = 4 414)**						**Girls **2** (** ***n*** **=4 276)**					
	Crude model* *			Adjusted model**			Crude model*			Adjusted model**			Crude model*			Adjusted model**		
	**cPR**	**95 CI**	**p**	**aPR**	**95 CI**	p	**cPR**	**95 CI**	***p***	aPR	**95 CI**	p	**cPR**	**95 CI**	***P***	**aPR**	**95 CI**	p
**physical punishment by father or mother**																		
No	Ref.			Ref			Ref			Ref			Ref			Ref		
Sí	0.81	0.73;0.91	<0.001	0.82	0.73;0.91	<0.001	0.70	0.58;0.83	<0.001	0.69	0.58;0.83	<0.001	0.92	0.80;1.06	0.254	0.93	0.81;1.06	0.278

*PR* Prevalence ratio (c = crude, a = adjusted), 95% CI = 95% confidence interval

^*^Generalized crude linear family model and Link Log Poisson option. Results are presented as prevalence ratio (cPR)

^**^Generalized adjusted linear family model and Link Log Poisson option. Results are presented as prevalence ratio (aPR)

For the whole analysis the complex sampling of the study was considered using the commands (svy)

^1^The overall model was adjusted for child’s sex, witness of violence, socioeconomic status, marital status, mother’s age, education level, and language, number of children, mother’s moderate-to-severe depressive symptoms and whether she works

^2^The boys and girls models were adjusted for socioeconomic status, marital status, mother’s age, education level, mother’s language, number of children, mother’s moderate-to-severe depressive symptoms and whether she works

In the crude model for boys, it was found that those who experienced physical punishment from their father and/or mother had a 30% lower likelihood of EVC compared with those who were not subjected to it (cPR 0.70 95% CI 0.58;0.83 *p* < 0.001). After adjusting for socioeconomic level, witnessing violence, mother’s marital status, age, education level, maternal language, number of children, moderate-to-severe depressive symptoms in the mother, and her occupation, a 31% lower likelihood of EVC was found (aPR 0.69 95% CI 0.58;0.83 *p* < 0.001) (Table 4).

Otherwise, in the crude model for girls, it was found that those who experienced physical punishment by their father and/or mother had an 8% lower likelihood of EVC compared with those who were not subjected to it (cPR 0.92 95% CI 0.80;1.06 *p* = 0.254), with no significant association. After adjusting for socioeconomic level, witnessing violence, mother’s marital status, mother’s age, education level, maternal language, number of children, moderate-to-severe depressive symptoms in the mother, and her occupation, a 7% lower likelihood of EVC was found (aPR 0.93 95% CI 0.81;1.06 *p* = 0.278), with no significant association (Table 4).

## Discussion

The study identified an association between physical punishment exerted by the father and/or mother and decreased EVC in boys aged 9–36 months. However, no such association was found in girls.

Regardless of the sex of the child, previous studies conducted during this early developmental stage have established an association between parental physical punishment and reduced verbal communication or language skills. Notably, a longitudinal study in the United States focusing on socioeconomically disadvantaged boys and girls employed the Bayley scale to assess verbal communication, revealing such an association [39]. In addition, the Fragile Families and Child Well-Being study—also a longitudinal investigation—found a connection between frequent maternal physical punishment and diminished receptive verbal ability in boys and girls within the same life stage [15]. However, our study’s findings indicate a significant association solely in boys, which has not been reported in other studies. Despite variations in research on the association between physical punishment, maltreatment, behavioral outcomes, and cognitive development, studies have recognized differences based on the child’s developmental stage and considered sex a modifying factor [19, 40]. Consequently, the present study contributes to evidence the association between physical punishment and EVC according to sex during the early developmental stages.

The finding of an association only in boys during this stage of life could be explained by the interaction of various individual factors at the child level, as well as interactions with parents, the family environment, and the normative, cultural, and social context.

Regarding individual factors, children at this stage are more vulnerable and dependent on their parents [41]. Based on their sex, they exhibit distinct genetic and biological characteristics such as: maturation rate, different neonatal neurodevelopment [8], the onset of word production, eye contact [4] and the initiation of use of gestures and imitation [5, 6]. Furthermore, there are sex-specific neuropsychological characteristics [10] such as stress response patterns [42–44] and higher levels of externalizing behavior problems in boys compared with girls, which may be related to imitating the of their parents’ behavior [45, 46]. Boys exhibit greater expression of anger and aggressive behavior when facing stressful situations [46], tending to reject the disciplinary methods chosen by their parents [47]. Alternatively, girls tend to internalize their response to physical punishment or maltreatment [42, 46].

Interaction factors with parents and the environment include parents’ expectations even before the child’s birth, as well as gender-based expected behaviors. Boys are more exposed to severe and frequent physical punishment compared with girls [48–50]. A greater number of family members and constant changes in family composition are associated with increased use of physical punishment by parents [51]. In addition, parents who have been subjected to physical punishment and/or maltreatment are influenced in their approach to correcting the behavior of their children [19].

Normative, cultural, and social context factors demonstrate that, particularly in developing countries, living in urban areas with more social problems related to stress levels, depression, and increased use of psychoactive substances leads to a greater employment of physical punishment as a means of disciplining children [51]. In these countries, boys are also more exposed to physical punishment than girls, as a form of discipline by their parents [41, 52]. This can be explained by the perception that boys are the future providers for the family, leading to the application of moderate-to-severe physical punishments between the ages of 1 and 5 due to their high dependence and vulnerability during this stage [41]. In addition, in most cultures, the prevailing message is that boys should be strong and independent, whereas girls should be vulnerable [53].

Moreover, in middle-income countries, parental attitudes favoring corporal punishment as a means of correcting children are influenced by social and cultural norms [54].

Gender-related social norms dictate the methods of correction and education employed with children and may encompass physical punishment as well as the dynamics of sex-based parent–child relationships (father, mother, son, and daughter). These gendered interaction norms provide daughters with protection that sons do not receive, as being a boy increases the likelihood of experiencing physical punishment from the father and/or mother [55].

Besides, no significant association has been found between physical punishment by the father and/or mother and reduced EVC in girls. This could be attributed to the fact that the most commonly utilized disciplinary method with girls might be psychological violence [40] and/or that girls tend to internalize their response to physical punishment or maltreatment [42, 46]. It is possible that even though a significant impact has not been observed in girls during this early stage, they may experience consequences in later stages of life, which may become evident as they grow older [42].

This study exhibits the strength of national representativeness, being one of the pioneering investigations into the association between physical punishment and EVC during early childhood, focusing on sex, within a developing country. However, several limitations should be acknowledged. Firstly, being a cross-sectional study, it allows for the identification of associations, but the temporal sequencing remains unclear. The reporting of child correction methods relies solely on maternal accounts, introducing the possibility of underreporting of physical punishment due to the sole-informant nature. Moreover, the precise nature of the physical punishment (e.g., open-ended strikes on specific body parts, such as buttocks, or the use of belts) as well as its severity and frequency, is not explicitly delineated. Comparisons with other studies are challenging due to the lack of standardized descriptions of physical punishment. While many studies consider punishments imposed by parents within the past 12 months, information regarding severity and intensity is not consistently captured. Furthermore, it is crucial to consider the unique characteristics of different studies and their contextual and cultural variations. In addition, it is plausible, albeit to a lesser degree, that children may experience physical punishment from other family members besides their parents.

ENDES did not measure certain variables, including child temperament, other forms of child maltreatment (such as sexual aggression or others), drug and/or alcohol consumption of the mother and or partner, and frequency and intensity of use. Information regarding father and mother involvement in verbal stimulation of the child, maternal intelligence level were also not included, although maternal education level was utilized. Another potential limitation was the presence of memory and social desirability biases, as self-reported responses from participants may have resulted in the omission of information from the past two weeks or could have been influenced by the inadvertent attitude of the interviewer.

## Conclusions

In children aged 9 to 36 months, this study revealed an association between physical punishment and reduced EVC in boys, whereas no such association was found in girls. The research reported a prevalence of physical punishment by parents of 16.7% in boys and 16.0% in girls. Concurrently, EVC was reported in 32.6% of boys and 40.5% of girls.

## Data Availability

This study entails a secondary analysis of the 2018–2019 ENDES, the data is freely available at https://proyectos.inei.gob.pe/microdatos/.
